# The association between time to antibiotics and relevant clinical outcomes in emergency department patients with various stages of sepsis: a prospective multi-center study

**DOI:** 10.1186/s13054-015-0936-3

**Published:** 2015-04-29

**Authors:** Bas de Groot, Annemieke Ansems, Daan H Gerling, Douwe Rijpsma, Paul van Amstel, Durk Linzel, Piet J Kostense, Marianne Jonker, Evert de Jonge

**Affiliations:** Leiden University Medical Center, Albinusdreef 2, 2300 RC Leiden, the Netherlands; Rijnstate Hospital, Wagnerlaan 55, 6815 AD Arnhem, the Netherlands; VU Medical Center, Boelelaan 1117, 1081 HV Amsterdam, the Netherlands

## Abstract

**Introduction:**

In early sepsis stages, optimal treatment could contribute to prevention of progression to severe sepsis. Therefore, we investigated if there was an association between time to antibiotics and relevant clinical outcomes in hospitalized emergency department (ED) patients with mild to severe sepsis stages.

**Methods:**

This is a prospective multicenter study in three Dutch EDs. Patients were stratified into three categories of illness severity, as assessed by the predisposition, infection, response, and organ failure (PIRO) score: PIRO score 1 to 7, 8 to 14 and >14 points, reflected low, intermediate, and high illness severity, respectively. Consecutive hospitalized ED patients with a suspected infection who were treated with intravenous antibiotics were eligible to participate in the study. The primary outcome measure was the number of surviving days outside the hospital at day 28 which was used as an inverse measure of hospital length of stay (LOS). The secondary outcome measure was 28-day mortality, taking into account the time to mortality.

Multivariable Cox regression analysis was used to estimate the association between time to antibiotics and the primary and secondary outcome measures corrected for confounders, including appropriateness of antibiotics and initial ED resuscitation, in three categories of illness severity.

**Results:**

Of the 1,168 included patients, 112 died (10%), while 85% and 95% received antibiotics within three and six hours, respectively. No association between time to antibiotics and surviving days outside the hospital or mortality was found. Only in PIRO group 1 to 7 was delayed administration of antibiotics (>3 hours) associated with an increase in surviving days outside the hospital at day 28 (hazard ratio: 1.46, 95% confidence interval: 1.05 to 2.02 after correction for potential confounders).

**Conclusions:**

In ED patients with mild to severe sepsis who received antibiotics within six hours after ED presentation, a reduction in time to antibiotics was not found to be associated with an improvement in relevant clinical outcomes.

**Electronic supplementary material:**

The online version of this article (doi:10.1186/s13054-015-0936-3) contains supplementary material, which is available to authorized users.

## Introduction

The association between time to antibiotics and relevant clinical outcomes has been extensively studied [1-9], especially in patients with severe sepsis and septic shock admitted to the intensive care unit (ICU). In three large retrospective studies with overall mortality ranging from 30 to 47%, delayed administration of antibiotics was associated with increased mortality [7-9], which has led to the advice in the Surviving Sepsis Campaign (SSC) to administer antibiotics within three hours in septic emergency department (ED) patients [10]. However, in one prospective study with excellent control for quality of hemodynamic resuscitation and a relatively low mortality of 19%, time to antibiotics was not found to be associated with mortality in septic shock patients, suggesting that hemodynamic resuscitation is more important than early administration of antibiotics. In this study, only administration of antibiotics before the onset of shock was associated with improved outcome [11]. The impact of early administration has never been studied prospectively in earlier sepsis stages, that is, before the onset of acute organ failure. In these patients progression to severe sepsis, which occurs in approximately 22% [12], could still be prevented and hemodynamic resuscitation is less important.

In addition to the association with mortality, several retrospective studies have suggested that early administration of antibiotics reduces hospital lengths of stay (LOS), potentially having an enormous impact on hospital finances and patient comfort and satisfaction [1,2,13,14]. However, the retrospective design of these studies made them prone to information bias, especially in the measurement of time to antibiotics, and limited the possibility to control for the potential confounding effect of quality of initial resuscitation. Furthermore, LOS has important interactions with mortality. For example, it may well be that most severely ill patients received antibiotics early. In these patients, hospital LOS may well be decreased due to early mortality, and not by beneficial effects of early administration of antibiotics.

### Importance

Presently, no studies have prospectively investigated the association between time to antibiotics and relevant outcomes in earlier sepsis stages. Given the high incidence of ED patients with uncomplicated sepsis who have a substantial chance to progress to severe sepsis [12], and the financial burden associated with severe sepsis [15,16], it would be important to explore if a measure as simple as early administration of antibiotics decreases hospital LOS, and thereby contributes to a reduction in healthcare-related costs [17].

### Goals of this investigation

The purpose of the present study was to prospectively study the association between time to antibiotics and hospital LOS and 28-day mortality in ED patients with a suspected infection, in three categories of disease severity, taking into account the quality of initial ED treatment (including appropriateness of antibiotics and hemodynamic resuscitation) and the confounding effect of mortality on hospital LOS.

## Methods

### Study design and setting

This was a prospective observational cohort study conducted in the EDs of three Dutch hospitals: the Leiden University Medical Center (LUMC; approximately 30,000 visits/year), the Rijnstate hospital (RH; an urban hospital with approximately 30,000 visits/year), and the VU University Medical Center (VUMC; approximately 30,000 visits/year). Patients were included from 1 June 2011 to 1 April 2013 in the LUMC, from 1 March 2012 to 1 April 2013 in the RH, and from 1 April 2012 to 1 April 2013 in the VUMC.

The study was approved by the medical ethics committee of the Leiden University Medical Center, who waived the need for individual informed consent as this was a purely observational study (P 12.014). In the Netherlands, approval of one ethical body is enough to perform a study in multiple centers.

### Selection of participants

All consecutive ED patients aged 17 years and older, with suspected infection and triage category (Manchester triage system, [18]) yellow, orange, or red, who were admitted to the hospital and treated with intravenous antibiotics were included by the triage nurse or the nurse or physician who took care of the patient. Triage categories blue and green were excluded because most of these patients were expected to be at very low risk for mortality or hospital admission (for example patients with a simple pharyngitis). Any sign that triggered the triage nurse and treating physician to suspect an infection was suitable (fever, coughing, erythema, and so forth). Patients who appeared to have no infection according to their final hospital discharge letter were excluded (for example, those with pulmonary embolus or auto-immune and hematologic disorders presenting with fever).

### Data collection

In all participating hospitals, the same SSC-based quality improvement program was used, in which a standard screening procedure was followed to optimize sepsis recognition and early ED resuscitation and disposition to an adequate level of care (see Additional file 1): the triage or treating nurse put a patient sticker on a registration form if a patient had a suspected infection and triage category yellow, orange, or red. All nurses and/or physicians were informed about the data that had to be collected by means of oral presentations, posters, and flyers in the ED, and the registration form which contained the study protocol. Demographic and comorbidity data, relevant time points and dates, laboratory variables, triage categories and vital signs, type of antibiotics, amount and type of fluids (L), administered oxygen (L/min), disposition, and outcome variables were prospectively registered in the digital hospital information system Chipsoft Ezis (Chipsoft, Amsterdam, Netherlands) of each participating hospital. A medical student or registrar in emergency medicine subsequently transferred data from the electronic hospital information system to a web-based data collection file that was specifically developed for the present study (PromiseBasic, Leiden, Netherlands), and which automatically calculated the illness severity scores, time to antibiotics, and number of surviving days outside the hospital at 28 days after ED presentation. After the inclusion period, data of the three participating hospitals were transferred to one SPSS file (SPSS version 20.0, IBM, New York, USA).

Illness severity was assessed by the initial predisposition, infection, response, and organ failure (PIRO) score, which has been specifically developed and validated for the ED population as described in this study [19], and has also been validated for the Dutch ED setting [20]. The PIRO scores were calculated retrospectively so that the treating physicians were not aware of the score at the time of ED presentation. Missing values were counted as normal in the calculations of the PIRO score, similar as in the acute physiology and chronic health evaluation (APACHE) score [21]. A patient was considered to have a ‘do not resuscitate’ (DNR) status if the existing medical files already stated that the patient had a DNR code, or if it was decided at the time of ED presentation or during hospital admission.

Time to antibiotics was measured by subtraction of registration time at the ED desk from the registered time of antibiotic administration by the nurse. To test the accuracy of the registration of the time of antibiotic administration, the registered time was compared to the observed time of antibiotic administration in 53 included patients in the three hospitals in the following way: one of the doctors in the participating institutions was asked to register the exact time at which the antibiotics were administered to the ED patient on the registration form. Nurses were not informed about this procedure. After the inclusion period was finished, the observed start time of administration of the first antibiotics was compared to the registered time of antibiotic administration (see Additional file 2). Time as zero was taken as the time at ED registration.

The appropriateness of the initial dose of antibiotics administered in the ED was assessed in retrospect as is summarized in Additional file 3. In culture-positive patients, initial antibiotics were considered to be appropriate if the cultured microorganism could be a causative pathogen in relation to the clinical findings, and showed *in-vitro* sensitivity for the initial dose of the antimicrobial agent. *Staphylococcus epidermidis*, *Staphylococcus hominis*, and other coagulase negative staphylococci were considered to be contaminates (and therefore analyzed as if culture-negative), except in cases with endocarditis or infections caused by foreign bodies such as prostheses. An infectious disease specialist helped to evaluate and interpret the microbiological data. Culture-negative patients were also included in the analysis because for the ED physician it is unknown at time of presentation if cultures will become positive, and these patients might have had an infection.

In culture-negative patients, the initial antibiotics prescribed in the ED were considered to have been effective when patients recovered without the need to change to other antibiotics because the patient clinically deteriorated. If patients died during hospital admission, antibiotics were considered to have been effective if the initial antibiotics were administered according to institutional protocol for antibiotic therapy for specific infections.

### Outcome measures

The primary outcome measure was the number of surviving days outside the hospital at day 28 after ED presentation. For example, if a patient would have been discharged alive after five days hospital admission, the number of surviving days outside the hospital would be 23 days. If the patient would have died in the hospital after five days of admission, the number of surviving days outside the hospital would be zero.

In this way the potential confounding effect of hospital mortality on hospital LOS is prevented. For example, if delayed treatment with antibiotics would result in prolonged hospital stay, this effect could be masked by increased early mortality. The endpoint ‘surviving days outside the hospital at day 28’ is analogous to the often used endpoint ‘ventilator free days at day 28’ in studies in ICU patients [22].

A secondary outcome measure was survival time up to 28 days after initial ED presentation. Patients were followed up on until 28 days after the initial ED presentation. Patients who were discharged from the hospital within 28 days were contacted by telephone to find out their survival status at 28 days.

### Data analysis

Data were presented as mean (SD) if normally distributed, and median (interquartile range (IQR)) if skewed. Cox regression analysis was used to assess the association between time to antibiotics and number of surviving days outside the hospital at day 28 because this is a non-parametric modeling method, requiring few assumptions about linearity, normality, or censored data.

It has been reported that treatment benefits may increase with illness severity, while others have suggested that only patients with intermediate illness severity benefit, meaning that patients with low or high disease severity will survive or die, respectively, regardless of the provided treatment; some therapies may even be harmful in low risk populations [23-25]. Because of the many potential confounders and interactions, we chose to do the analyses in three separate groups with low (PIRO score 1 to 7), moderate (PIRO score 8 to 14), or high initial illness severity (PIRO score >14), rather than adding interaction terms in the model, because this requires less assumptions. Within each PIRO category, adjustment by PIRO score was performed.

Time to antibiotics was divided into three categories because the association between time to antibiotics and outcome was not expected to be linear, based on the combination of observations in previous studies [7-9]. Categories were administration within one hour, between one and three hours, and after three hours, because these categories were considered most relevant for the ED setting.

An association model [26], as opposed to a prediction model, was constructed per PIRO group to describe the association between time to antibiotics and outcome as accurate as possible. First, each of the following predefined potential confounders were added separately into the starting model with time to antibiotics and outcome: patient variables (the separate P, I, R, and O scores) in addition to the use of antibiotics, statins, or β-blockers prior to ED presentation, because previous studies showed that these might affect outcome [1,27-29]; ED treatment variables (amount of fluids, oxygen, and appropriateness initial antibiotics); and disposition to ward or ICU (or medium care unit (MCU)). The participating hospital was also put in the model as a separate variable to correct for structural differences among participating hospitals. The variable that resulted in the largest change of the regression coefficient of the association between the time to antibiotics and outcome was then added to the model, which subsequently became the new starting model. This procedure was repeated until addition of a new predefined potential confounder resulted in a change of the regression coefficient of the primary association of interest of less than 10%, which was no longer considered relevant.

Hazard ratios ((HR) and 95% confidence intervals (CI) for the association between time to antibiotics (antibiotics within one hour was set as the reference) and outcomes were calculated and reported for the crude and corrected models. Although not the explicit purpose of the present study, HRs of the confounders of the corrected models were also reported because these could help in generating hypothesis for future studies. The *P* values of these confounders were therefore reported, but were not used to construct the model. All data were analyzed using SPSS software (SPSS 20.0, IBM, New York, USA).

### Sensitivity analysis

Because the appropriateness of antibiotics, the absence of a positive culture, and DNR status could have an important effect on the primary association of interest, a sensitivity analyses were performed. First, the aforementioned analysis was performed in patients who received appropriate and inappropriate antibiotics in two separate models. Second, the same analysis was performed with and without forced entry of DNR status. Third, the same analysis was performed only in culture-positive patients. Fourth, we also performed the analysis with forced entry of all potential predefined variables, to be sure that the model was not affected by the stepwise procedure itself.

### Sample size calculations

For the outcome ‘number of surviving days outside the hospital at day 28 after ED presentation’, the present study had a power of 80%, calculated *a priori* to detect a difference in outcome (α = 0.05) of one day between a group with time to antibiotics below or above the median time to antibiotics. The expected number of surviving days outside the hospital at day 28 was 23, and was derived from the study of Houck *et al*. [1]. In this calculation, the skewed distribution of the number of surviving days outside the hospital was taken into account. It was calculated that approximately 400 inclusions per PIRO category were needed. The rule of thumb that approximately 10 events per potential confounder was needed for sufficient power was taken into account, in order to set the number of confounders that could be adjusted for in the analysis, with 28-day mortality as outcome measure.

## Results

### Patient characteristics and inclusion

**Figure 1:**
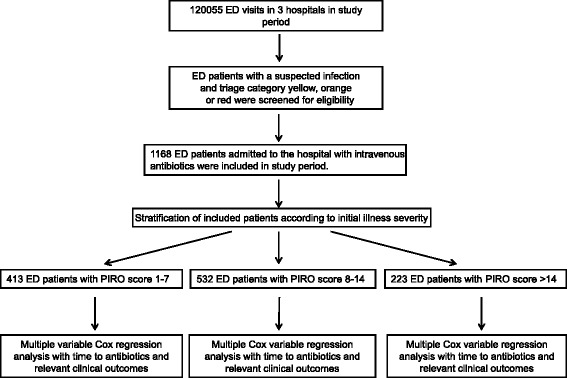
Patient inclusion and flow through study. Illness severity was expressed as Predisposition, Infection, Response, and Organ failure (PIRO) score.

Figure 1 shows a diagram of patient inclusion and flow through the study. A total of 1,168 patients were included: 413 patients in PIRO category 1 to 7, 532 patients in PIRO category 8 to 14, and 223 patients in PIRO category >14. Patient characteristics are shown in Table 1 as a function of illness severity. Time to antibiotics decreased with increasing PIRO category. However, within one PIRO category there was no association between PIRO score and time to antibiotics (minutes); regression coefficients (CI) for linear regression were −12.38 (−35.05 to 10.30) for PIRO 1 to 7, −10.21 (−25.90 to 5.48) for PIRO 8 to 14 and 8.44 (−12.36 to 29.25) for PIRO >14.

**Table 1:** **Patient characteristics as a function of illness severity as indicated by PIRO category**

	**Total cohort (n = 1,168)**	**PIRO 1 to 7 (n = 413)**	**PIRO 8 to 14 (n = 532)**	**PIRO >14 (n = 223)**
**Demographics**				
Age, mean (SD)	62 (17)	54 (16)	64 (17)	72 (14)
Male sex (%)	650 (56)	211 ((51)	295 (55)	144 (65)
**Comorbidities, n (%)**				
COPD	191 (16)	23 (6)	101 (19)	67 (30)
Heart disease	104 (9)	24 (6)	51 (10)	29 (13)
Liver disease (**1**)	42 (4)	11 (3)	13 (2)	18 (13)
Renal disease (**1**)	172 (15)	55 (13)	72 (14)	45 (20)
Malignancy−	137 (12)	41 (10)	57 (11)	39 (17)
Malignancy+ (**2**)	131 (11)	32 (8)	63 (12)	36 (16)
Immune-compromised (**1**)	410 (35)	159 (38)	163 (31)	88 (39)
Nursing home (**1**)	79 (7)	4 (1)	29 (5)	46 (21)
DNR status (**6**)	277 (24)	40 (10)	133 (25)	104 (47)
**Medication use at ED presentation, n (%)**				
β-Blocker/Ca-antagonist (**13**)	348 (30)	103 (25)	169 (32)	76 (34)
Statins (**11**)	284 (33)	81 (20)	142 (27)	61 (27)
Antibiotic use (**10**)	306 (26)	99 (24)	138 (26)	69 (69)
**Vital signs at ED presentation, mean (SD)**				
Respiratory rate (per minute) (**373**)	26 (8)	18 (4)	27 (7)	30 (8)
Oxygen saturation (%) (**60**)	95 (5)	97 (3)	94 (6)	93 (6)
Heart rate (per minute) (**40**)	109 (20)	105 (18)	109 (20)	116 (23)
Systolic BP (mmHg) (**186**)	133 (27)	130 (24)	137 (27)	129 (30)
Diastolic BP (mmHg) (**187**)	76 (17)	75 (15)	77 (17)	72 (19)
Temperature (°C) (**32**)	38.7 (2.3)	38.6 (1.8)	38.9 (2.8)	38.7 (1.2)
Altered mental status	208 (18)	40 (10)	100 (19)	68 (30)
Febrile chills	278 (24)	126 (31)	114 (21)	38 (17)
**Laboratory, median (IQR)**				
Lactate (mmol/L) (**202**)	1.8 (1.3-2.5)	1.6 (1.2-2.1)	1.7 (1.3-2.4)	2.2 (1.6-4.0)
Creatinine (μmol/L) (**9**)	87 (66–118)	81 (65–102)	83 (64–116)	110 (82–156)
Urea (mmol/L) (**31**)	6.8 (5–10.1)	5.6 (4.4-7.6)	6.8 (5.0-9.9)	10 (7.7-14.4)
Platelets (10^12^/mm^3^) (**24**)	210 (155–282)	209 (164–279)	218 (163–287)	182 (117–270)
Bilirubin (μmol/L) (**404**)	12 (8–18)	12 (9–18)	12 (8–18)	13 (9–24)
**Suspected site of infection, n (%)**				
Lung	617 (513	105 (25)	326 (61)	186 (83)
UTI	335 (29)	113 (27)	145 (27)	77 (35)
Abdominal	199 (17)	89 (22)	83 (16)	27 (12)
Skin	97 (8)	63 (15)	27 (5)	7 (3)
Neurological	33 (3)	10 (1)	16 (3)	7 (3)
Other	192 (16)	86 (21)	86 (16)	20 (9)

Data are presented as mean (SD) if normally distributed, or as median (IQR) if rightly skewed. Categorical data are presented as number (%). Percentages of suspected sites of infection do not add up to a 100% because some patients had multiple suspected sites of infection at the time of ED presentation. Missing values are shown in bold between brackets for every variable.

BP, blood pressure; COPD, chronic obstructive pulmonary disease; DNR status, do not resuscitate status; ED, emergency department; IQR, interquartile range; Malignancy−, malignancy without metastases; Malignancy+, malignancy with metastases and hematologic malignancy; MV, missing value; PIRO, Predisposition, Infection, Response, and Organ failure score; SD, standard deviation; UTI, urinary tract infection.

A total of 85% and 95% of the patients received antibiotics within three and six hours, respectively. The accuracy of registration of time to antibiotics is shown in Additional file 2. Excellent correlation was found between the registered time to antibiotics and time to antibiotics by an independent observer (N = 53, r^2^: 0.926, regression coefficient (SEM): 0.98 (0.04), constant: −0.72 minutes). Overall 28-day mortality in the present cohort was 10%, ranging from 2% in PIRO group 1 to 7 to 23% in PIRO group >14.

### Association between time to antibiotics and relevant clinical outcomes

The association between time to antibiotics with potential confounders, and surviving days outside the hospital at day 28 (primary endpoint) and 28-days mortality is shown in Tables 2 and 3.

**Table 2:** **Number of surviving days outside the hospital at day 28 as a function of time to antibiotics in ED patients with a suspected infection in three categories of illness severity**

	**Crude model HR (95% CI)**	**Corrected model HR (95% CI)**	***P *** **(Corrected model)**
**PIRO group 1 to 7**			
**Antibiotics <1 hour (reference category)**	1	1	0.02^a^
Antibiotics 1–3 hours	0.84 (0.66-1.06)	1.03 (0.78-1.36)	0.824
Antibiotics >3 hours	1.11 (0.84-1.46)	1.46 (1.05-2.02)	0.023
Type of hospital (academic versus urban)		0.635 (0.486-0.831)	0.001
P score		1.16 (1.04-1.30)	0.008
O score		1.03 (0.96-1.12)	0.408
Statin use		1.32 (1.02-1.70)	0.033
Appropriateness of antibiotics		0.61 (0.46-0.81)	0.001
Amount of oxygen (L/min)		1.01 (0.99-1.04)	0.301
Amount of fluids (L/ED stay)		1.23 (1.06-1.43)	0.005
**PIRO group 8 to 14**			
**Antibiotics <1 hour (reference category)**	1	1	0.984^a^
Antibiotics 1–3 hours	0.85 (0.70-1.02)	1.02 (0.83-1.25)	0.863
Antibiotics >3 hours	0.79 (0.60-1.04)	1.02 (0.75-1.38)	0.910
Type of hospital (academic versus urban)		0.54 (0.44-0.66)	<0.001
P score		1.05 (1.00-1.11)	0.05
O score		1.06 (1.00-1.13)	0.037
Appropriateness of antibiotics		0.76 (0.61-0.93)	0.008
Amount of oxygen (L/min)		1.02 (1.00-1.04)	0.015
Amount of fluids (L/ED stay)		1.03 (0.92-1.16	0.616
**PIRO group >14**			
**Antibiotics <1 hour (reference category)**	1	1	0.361^a^
Antibiotics 1–3 hours	1.00 (0.75-1.32)	1.16 (0.86-1.58)	0.366
Antibiotics >3 hours	0.89 (0.57-1.40)	1.40 (0.84-2.34)	0.194
Type of hospital (academic versus urban)		0.69 (0.50-0.96)	0.025
P score		1.10 (1.00-1.22)	0.041
O score		1.09 (1.02-1.17)	0.017
Statin use		0.84 (0.61-1.15)	0.277
Antibiotics use		1.04 (0.77-1.41)	0.789
Appropriateness of antibiotics		0.70 (0.51-0.95)	0.024
Amount of oxygen (L/min)		1.03 (1.01-1.05)	0.014
Amount of fluids (L/ED stay)		1.14 (1.01-1.29)	0.042

Cox regression analysis was performed with time to antibiotics divided into three categories, corrected for possible predefined confounders: PIRO score; antibiotic, β-blocker, or statin use prior to ED presentation; efficacy of antibiotics; amount of fluids (L); amount of oxygen (L/min) in the ED; and type of hospital (academic versus urban hospital). Hazard ratio >1 indicates a larger number of days outside the hospital at day 28 after ED presentation compared to the reference category of time to antibiotics within one hour. ^a^Overall *P* value for categories of time to antibiotics. The *P* values were not used to construct the model.

CI, Confidence interval; ED, Emergency department; HR, Hazard ratio; ICU, Intensive care unit; and PIRO, Predisposition, Infection, Response, and Organ failure score (as a measure of illness severity).

**Table 3:** **The hazard for 28-day mortality as a function of time to antibiotics in ED patients with suspected infection in three categories of illness severity**

	**Crude model (HR (95% CI)**	**Corrected model (HR (95% CI)**	***P *** **(Corrected model)**
**PIRO group 1 to 7 (n = 413)**			
**Antibiotics <1 hour (reference category)**	1	1	0.422^a^
Antibiotics 1–3 hours	0.95 (0.17-5.18)	2.55 (0.36-18.25)	0.352
Antibiotics >3 hours	1.98 (0.36-10.78)	5.31 (0.43-68.16)	0.191
Type of hospital (academic versus urban)		0.06 (0.007-0.48)	0.008
P score		2.53 (1.41-4.56)	0.002
Appropriateness of antibiotics		0.33 (0.06-1.66)	0.180
Amount of oxygen (L/min)		1.17 (1.02-1.35)	0.028
Amount of fluids (L/ ED stay)		1.65 (0.76-3.59)	0.205
**PIRO group 8 to 14 (n = 532)**			
**Antibiotics <1 hour (reference category)**	1	1	0.676^a^
Antibiotics 1–3 hours	1.11 (0.62-1.99)	1.25 (0.62-2.31)	0.488
Antibiotics >3 hours	0.65 (0.22-1.90)	0.86 (0.28-2.63)	0.786
Type of hospital (academic versus urban)		0.35 (0.19-0.68)	0.002
P score		1.29 (1.14-1.46)	<0.001
R score		0.74 (0.62-0.89)	0.001
O score		1.28 (1.10-1.50)	0.002
Amount of oxygen (L/min)		1.04 (1.00-1.09)	0.067
**PIRO group >14 (n = 223)**			
**Antibiotics <1 hour (reference category)**	1	1	0.978^a^
Antibiotics 1–3 hours	1.10 (0.62-1.97)	0.99 (0.53-1.87)	0.983
Antibiotics >3 hours	0.93 (0.36-2.43)	1.11 (0.40-3.08)	0.849
Type of hospital (academic versus urban)		1.57 (0.83-3.00)	0.166
O score		1.15 (1.00-1.34)	0.056
β-blocker use		0.96 (0.51-1.79)	0.892
Appropriateness of antibiotics		0.35 (0.19-0.62)	<0.001
Amount of oxygen (L/min)		1.01 (0.98-1.04)	0.414
Amount of fluids (L/ED stay)		0.88 (0.65-1.20)	0.425

Cox regression analysis was performed with time to antibiotics divided into three categories. In the corrected model regression coefficients were corrected for possible predefined confounders: PIRO score; β-blocker, statin, and antibiotic use prior to ED presentation; appropriateness of initial antibiotics in the ED; amount of fluids (L); amount of oxygen (L/min) in the ED; and hospital (academic versus urban hospital). ^a^Overall *P* value for categories of time to antibiotics. The *P* values were not used to construct the model.

CI, Confidence interval; ED, Emergency department; HR, Hazard ratio; ICU, Intensive care unit; PIRO, Predisposition, Infection, Response, and Organ failure score (as a measure of illness severity).

In the analysis, administration of antibiotics within an hour was set as reference category to which the other two categories were compared. For example, a HR larger than 1 in the category ‘time to antibiotics >3 hours’ indicates that administration of antibiotics after three hours is associated with a larger number of surviving days outside the hospital at day 28 (In Table 2), or a larger hazard for mortality (in Table 3). An HR below 1 indicates the opposite. In the present cohort with relatively low mortality and a small range of time to antibiotics, a reduction in time to antibiotics was not found to be significantly associated with an improvement of relevant clinical outcomes, that is, the HRs were not significantly different from 1. However, for the PIRO category with lowest illness severity (1 to 7), time to antibiotics under three hours was associated with a larger number of surviving days outside the hospital at day 28 after ED presentation. No association was found between time to antibiotics and survival time censored at 28 days, irrespective of severity of illness by PIRO category.

In Table 4 the relevant treatment aspects and outcomes per PIRO category were shown as a function of the three categories of time to antibiotics. It can be seen that the percentage of appropriateness of antibiotics is not higher in the group with administration of antibiotics after three hours. In addition, the relevant clinical outcomes are also similar among time to antibiotics categories.

**Table 4:** **Treatment variables and relevant clinical outcomes per PIRO category as a function of timing of antibiotics**

	**Antibiotics <1 hour (reference category)**	**Antibiotics 1–3 hours**	**Antibiotics >3 hours**
PIRO 1 to 7 (N = 413)	n = 101	n = 211	n = 101
**ED treatment**			
Number of SSC goals achieved, mean (SD) **(9)**	3.3 (1.0)	3.7 (0.9)	2.7 (0.9)
Time to antibiotics (minutes), median (IQR) **(1)**	41 (32–51)	110 (85–141)	295 (216–516)
Initial antibiotics appropriate, n (%) **(11)**	84 (84)	182 (86)	73 (72)
Administered fluids (L), median (IQR)	1.0 (0.5-1.5)	1.0 (0.5-1.5)	0.5 (0.5-1.0)
Administered oxygen (L/min), median (IQR) **(53)**	2.0 (0.0-3.0)	0 (0.0-3.0)	0 (0.0-3.8)
**Disposition/outcomes**			
MCU/ICU admission, n (%) **(18)**	7 (7)	7 (209)	2 (2)
Total hospital LOS (days), median (IQR)	5 (3–8)	4 (2–8)	6 (3–12)
Number of surviving days outside hospital at day 28, median (IQR)	23 (20–25)	23 (19–26)	21 (15–25)
28-day mortality, n (%) **(7)**	2 (2)	4 (2)	4 (2)
**PIRO 8 to 14 (N = 532)**	n = 217	n = 249	n = 66
**ED treatment**			
Number of SSC goals achieved, mean (SD) **(9)**	3.7 (1.1)	3.7 (0.9)	3.0 (0.9)
Time to antibiotics (minutes), median (IQR) **(1)**	38 (24–48)	102 (78–133)	243 (201–367)
Initial antibiotics appropriate, n (%) **(11)**	166 (76)	183 (73)	50 (76)
Administered fluids (L), median (IQR)	1.0 (0.5-1.6)	1.0 (0.5-1.5)	1.0 (0.5-1.5)
Administered oxygen (L/min), median (IQR) **(53)**	2.0 (1.0-5.0)	3.0 (0.0-5.0)	3.0 (0.0-5.0)
**Disposition/outcomes**			
MCU/ICU admission, n (%) **(18)**	29 (13.4)	23 (9)	2 (3)
Total hospital LOS (days), median (IQR)	6 (4–10)	6 (3–9)	5 (3–9)
Number of surviving days outside hospital at day 28, median (IQR)	21 (15–24)	22 (14–24)	22 (18–25)
28-day mortality, n (%) **(7)**	20 (3)	26 (10)	4 (6)
**PIRO >14 (N = 223)**	n = 113	n = 87	n = 23
**ED treatment**			
Number of SSC goals achieved, mean (SD) **(9)**	4.0 (1.1)	3.8 (1.0)	3.0 (0.7)
Time to antibiotics (minutes), median (IQR) **(1)**	33 (23–33)	95 (81–126)	295 (218–499)
Initial antibiotics appropriate, n (%) **(11)**	81 (74)	61 (70)	18 (78)
Administered fluids (L), median (IQR)	1.0 (0.5-2.0)	1.0 (0.5-1.6)	0.6 (0.5-1.5)
Administered oxygen (L/min), median (IQR) **(53)**	5.0 (2.0-10.0)	3.0 (2.0-10.0)	3.0 (1.5-5.0)
**Disposition/outcomes**			
MCU/ICU admission **(18)**	33 (29)	15 (17)	4 (17)
Total hospital LOS (days), median (IQR)	7 (5–13)	8 (3–12)	7 (3–8)
Number of surviving days outside hospital at day 28, median (IQR)	18 (0–22)	17 (4–22)	20 (3–23)
28-day mortality, n (%) **(7)**	26 (23)	21 (24)	5 (22)

Data are presented as mean (SD) if normally distributed or as median (IQR) if rightly skewed. Categorical data are presented as number (%). Missing values are shown in bold between brackets for every variable.

ED, Emergency Department; ICU, Intensive Care Unit; IQR, interquartile range; LOS, Length of Stay; MCU, Medium Care Unit; PIRO, Predisposition, Infection, Response, and Organ failure; SD, standard deviation; SSC, Surviving Sepsis Campaign.

Although the present study did not aim to test if there were other potential predictors of relevant clinical outcomes, the associations between the confounders of the primary association of interest and the primary and secondary outcome measures were shown in Tables 2 and 3 because they may explain the lack of a significant association between time to antibiotics and relevant clinical outcomes. Other aspects of ED treatment, such as the amount of administered oxygen and fluids and the appropriateness of the initial dose of antibiotics administered in the ED, were associated with relevant clinical outcomes which may provide important directives for future studies.

#### Sensitivity analyses

First, the analyses were performed separately in ED patients who received appropriate and inappropriate antibiotics to investigate if the primary association of interest was different: the corrected HRs for surviving days outside the hospital at day 28 (antibiotics within one hour was set as reference) for the group who received appropriate antibiotics (n = 899) were 1.58 (95% CI: 0.865 to 2.87, *P* = 0.137) and 1.43 (95% CI: 0.614 to 3.34, *P* = 0.406) for patients who received antibiotics between one and three and after three hours, respectively. For patients who received inappropriate antibiotics (n = 258) the corrected HRs were 0.96 (95% CI: 0.48 to 1.91) and 0.31 (95% CI: 0.068 to 1.46). Analysis in the three separate PIRO groups yielded similar results. Similarly, analyses in only patients with positive cultures (n = 651) did not change the primary association of interest (data not shown). Furthermore, forced entry of all potential predictor variables to the model did not change the primary association of interest.

## Discussion

The main new finding of the present study is that a reduction in time to antibiotics was not found to be associated with an improvement of relevant clinical outcomes in our cohort of ED patients with mild to severe stages of sepsis. A reduction in time to antibiotics was also not found to be associated with an increase in number of surviving days outside the hospital at day 28 after ED presentation. Corresponding to the number of ventilator-free days in ICU-related studies [22], we chose this primary endpoint instead of hospital LOS to avoid confounding of the association between time to antibiotics and hospital LOS by mortality.

Our findings are in contrast to a study by Houck *et al*. of approximately 14,000 patients with a pneumonia, which found an increase in hospital LOS of 0.4 days with administration of antibiotics after four hours [1]. Several differences may explain the discrepancy. The retrospective design of the study is prone to information bias, especially with regard to the timing of antibiotics. Furthermore, it consisted of a population with only elderly patients with pneumonia, while in our study all adults with all sources of infection were included. Also, there was no control for appropriateness of the initial choice of antibiotics and amount of fluids. Several smaller retrospective studies that found an increased hospital LOS with administration of antibiotics beyond two to eight hours had similar methodological drawbacks [13,14].

Unexpectedly, we found that in the PIRO 1 to 7 group, administration of antibiotics after three hours was significantly associated with a larger number of surviving days outside the hospital. It is unlikely that delaying antibiotic treatment is beneficial for patients with infections. The most likely explanation for the unexpected association of delayed administration of antibiotics with shorter hospital stay is that administration of antibiotics is more often delayed in patients with less severe infections. In contrast, severely ill patients may be administered antibiotics shortly after admission to the ED. This confounding effect of severity of illness may not be completely adjusted for by including the PIRO score in the multivariable analysis, because in the low illness severity range (PIRO 1 to 7) the PIRO score has limited discriminative value, as shown in the study by Howell *et al*. [19]. In contrast to the study by Hranjec *et al*. in surgical ICU patients [30], the percentage of patients who received appropriate antibiotics in the ED was lower in the group with delayed administration of antibiotics. This could therefore not explain the association of delayed administration of antibiotics and the larger number of surviving days outside the hospital in PIRO category 1 to 7. Finally, in the sensitivity analyses the primary association of interest did not change, but the aforementioned unexpected finding was no longer significant. It cannot be excluded, however, that unknown confounders may be responsible for this unexpected finding.

In this prospective study in ED patients, a reduction in time to antibiotics was not found to be associated with a reduction in mortality. The most likely explanation for the discrepancy with the three large retrospective studies that showed that delayed administration of antibiotics is associated with increased mortality is the much lower overall mortality, that is, disease severity, in the present study. This suggests that, in less severely ill patients, timing of antibiotics is less important than other aspects of ED treatment, including appropriateness of antibiotics and initial resuscitation with fluids and oxygen. Although it should be emphasized that the present study was not designed to estimate the effect of other treatment variables on relevant clinical outcomes, it should be noted that several confounders of the primary association of interest, such as appropriateness of antibiotics, supplemental oxygen, and fluids, were significantly associated with the clinical endpoints of the present study. Interestingly, appropriateness of initially administered antibiotics was associated with lower mortality, but possibly at the expense of less surviving days outside the hospital. It may be hypothesized that these variables represent more important aspects of ED treatment compared to time to antibiotics, at least in study cohorts with relatively low mortality. Correspondingly, in a study by Rivers *et al*., early goal-directed therapy (EGDT) had an enormous impact on outcome in a study cohort with 47% mortality [31], while the ProCESS trial (Protocol-based Care for Early Septic Shock) showed no benefit of EGDT in a study cohort with a much lower mortality of around 20% [32], which is in line with the previous observations that treatment benefits depend on disease severity. Indeed, some effective therapies were shown to be less relevant or even harmful in low risk populations [23-25].

### Study strengths and weaknesses

The strengths of the present study include the prospective design, the adjustment for illness severity, the quantification of the accuracy of the registration of time to antibiotics, and excellent control for predefined potential confounders, including ED treatment and relevant medication. However, there are also some limitations.

First, in the most severely ill group (PIRO >14), there were fewer than 400 patients, therefore the power in this group might be relatively low. However, although we wanted to explicitly investigate the effect of illness severity on the association between time to antibiotics and relevant outcomes, in retrospect the illness severity (PIRO category) did not affect the association. Therefore, we also investigated all 1,168 included patients in a model with PIRO score as an interaction term with time to antibiotics in SPSS. In this model, the interaction term was not significant (*P* = 0.209), indicating that in retrospect we indeed could have analyzed all patients in one group. After having performed this analysis the main conclusion of the study still stands: no association between time to antibiotics and relevant clinical outcomes was found. In this analysis approximately three times more patients were analyzed than needed (*a priori* calculated sample size of 400). It is therefore unlikely that the absence of an association is attributed to limited power of the study. The relatively small number of deaths (secondary outcome) in PIRO group 1 to 7 may have resulted in less accurate effect size estimations. The HRs in this group with very mild disease severity should therefore be interpreted with caution.

Second, because many patients had negative cultures, the estimation of appropriateness of the initial choice of antibiotics may include inaccuracies. We believe, however, that the predefined flow diagram to decide if an antimicrobial agent was appropriate was the most objective way in culture-negative patients. Furthermore, similar strategies to interpret appropriateness of antibiotics were used in the most relevant previous studies [7-9,11], facilitating comparison of our results with those found in the literature. In addition, a realistic association between efficacy of antibiotics and relevant endpoints is also obtained by taking into account potential side effects related to antibiotic administration to patients who may not have needed antibiotics, such as those suffering from viral infections. More importantly, in clinical practice, many patients receive antibiotics while being culture-negative, either reflecting more localized infections like a pneumonia without bacteremia, or viral infections. These patients should be included to assess the overall benefit of antibiotics.

## Conclusions

In our study cohort of ED patients with mild to severe sepsis stages and overall mortality of 10%, a reduction in time to antibiotics was not found to be associated with an improvement in relevant clinical outcomes. Future studies should investigate if other aspects of ED treatment, especially appropriateness of initial antibiotics and initial resuscitation with fluids and oxygen, are more important in sepsis stages preceding septic shock.

## Key messages

- A reduction in time to antibiotics was not found to be associated with an improvement in hospital length of stay in emergency department patients with mild to severe sepsis stages.
- A reduction in time to antibiotics was not found to be associated with a reduction in 28-day mortality in emergency department patients with mild to severe sepsis stages.
- Future studies should investigate if other aspects of ED treatment, especially appropriateness of initial antibiotics and initial resuscitation with fluids and oxygen, are more important in sepsis stages preceding septic shock.

## Additional files

Additional file 1:
**Flow diagram of the quality improvement program used in the participating hospitals.**
Additional file 2:
**The association between the observed and registered time to antibiotics.**
Additional file 3:
**Flow diagram of the definition of the appropriateness of initial dose of antibiotics administered in the emergency department.**

## Abbreviations

APACHE: Acute physiology and chronic health evaluation
CI: Confidence interval
COPD: Chronic Obstructive Pulmonary Disease
DNR: Do Not Resuscitate
ED: Emergency department
EGDT: Early goal-directed therapy
HR: Hazard ratio
ICU: Intensive care unit
IQR: Inter quartile range
L: Liter
LOS: Lengths of stay
MCU: Medium Care Unit
PIRO: Predisposition, Infection, Response, and Organ failure
RC: Regression coefficient
SD: Standard deviation
SEM: Standard error of the mean
SSC: Surviving Sepsis Campaign
