# Supplementary material for: The association between time to antibiotics and relevant clinical outcomes in emergency department patients with various stages of sepsis: a prospective multi-center study
Source: Crit Care. 2015 Apr 29;19(1):194. doi: 10.1186/s13054-015-0936-3 (PMC4440486; doi:10.1186/s13054-015-0936-3)
Supplement: Additional file 1: — Flow diagram of the quality improvement program used in the participating hospitals. [file 13054_2015_936_MOESM1_ESM.doc]

**Does the patient have a suspected infection and triage category yellow, orange or red?**

**Nrse**

**Is there 1 alarming symptom or more potential alarming symtoms?**

**No**

**Re-evaluate patient regularly**

**Yes**

Inform physician and provide all vital signs

Give O_2_, give i.v. NaCl 0,9% 500 ml. Time:……..u.

Take 2 sets bloedcultures and and other cultures if indicated. Time:……..u.

Lab: Leuco’s, Diff., Hb, trombocytes, creatinine, urea, Na, K, ALAT, ASAT, ɣ-GT, AF,

bilirubine, amylase, CRP, lactate, troponin, INR/APTT. Consider bloodgas.

Ask physician what antibiotics should be administered.

Place urinary catheter if blood pressure syst. < 90 mmHg, MAP < 65 mmHg (ask physcian)

Give 1 to 1,5 liter NaCl 0,9% in 30 min.

Communicate suspected diagnosis with nurse

Consult infectious disease specialist if necessary

Check if all cultures are taken and if additional test

Are indicated.

Administer antibiotics directly based on focus

and institutional protocol. **Time:………….hrs**

Evaluate effect of treatment*

Evaluate labs

Search for signs of new onset organ failure**

**Therapy is not effective**

**Signs of organ failure**

**Consider**

**ICU consultation (pager 9182)**

**Admission ward**

**(Possible discharge home)**

Alarming symptoms

SO_2_ < 90%

Bloodpres. syst. < 90 mmHg

Bloodpres syst. > 40 mmHg gedaald

Skin signs/capillary refill > 2 sec.

Meningeal signs

Altered mental status

Petechiën

Potential alarming symptoms

Tachypnoe > 20/min

Tachycardia > 90/min

Temperature > 38 of < 36°C

Immuno-compromised

**POSSIBLE SEPSIS!**

Pneumonia (sputum culture)

Intra-abdominal infection

Urineweginfectie (urine culture)

Skin/bone/joint infection

Meningitis (liquor culture)

Implantant/line infection/TSS

Endocarditis

Other: i.e. malaria

SO_2_ > 90%, no signs of exhaustion

Bloodp. syst. > 90 mmHg, MAP > 65 mmHg. Time:… hr

Urine production > 0,5 ml/kg/hr

Normal mental status Tijd:….....u

**Remarks**:......................................................................

Respiratoiry insufficiency

Lactate > 4 mmol/L

Creatinine > 178 ųmol/L

INR >1,5 of APTT > 60 sec

Trombocytes < 100 10^9^/L

Bilirubin > 34 µmol/L

**Yes**

**Suspected**

**SEVERE SEPSIS!**

**F**

**O**

**C**

**U**

**S**

**Therapy is effective**

**No signs of organ failure**

**Nrse**

**Nrsee**

**Dr**

**Dr**

**Possible signs of new-onset organ failure****

**Targets for an effective therapy***
