# Supplementary figures and images for: The association between time to antibiotics and relevant clinical outcomes in emergency department patients with various stages of sepsis: a prospective multi-center study
Source: Crit Care. 2015 Apr 29;19(1):194. doi: 10.1186/s13054-015-0936-3 (PMC4440486; doi:10.1186/s13054-015-0936-3)

**Additional file 2.**


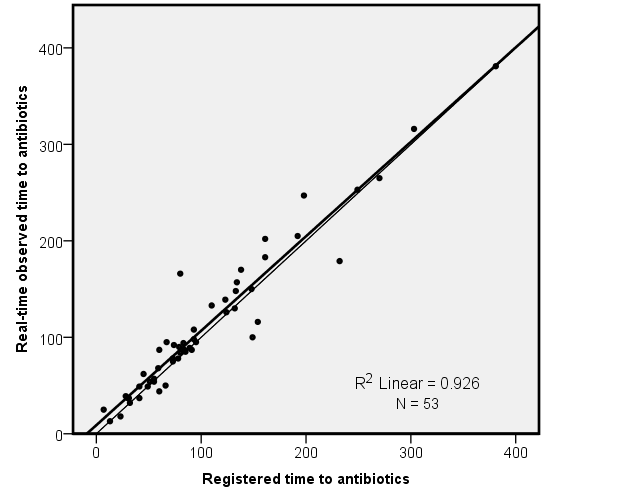

Supplement: Additional file 2: — The association between the observed and registered time to antibiotics. [file 13054_2015_936_MOESM2_ESM.doc]
