# Supplementary material for: The association between time to antibiotics and relevant clinical outcomes in emergency department patients with various stages of sepsis: a prospective multi-center study
Source: Crit Care. 2015 Apr 29;19(1):194. doi: 10.1186/s13054-015-0936-3 (PMC4440486; doi:10.1186/s13054-015-0936-3)
Supplement: Additional file 3: — Flow diagram of the definition of the appropriateness of initial dose of antibiotics administered in the emergency department. [file 13054_2015_936_MOESM3_ESM.ppt]

## Slide 1
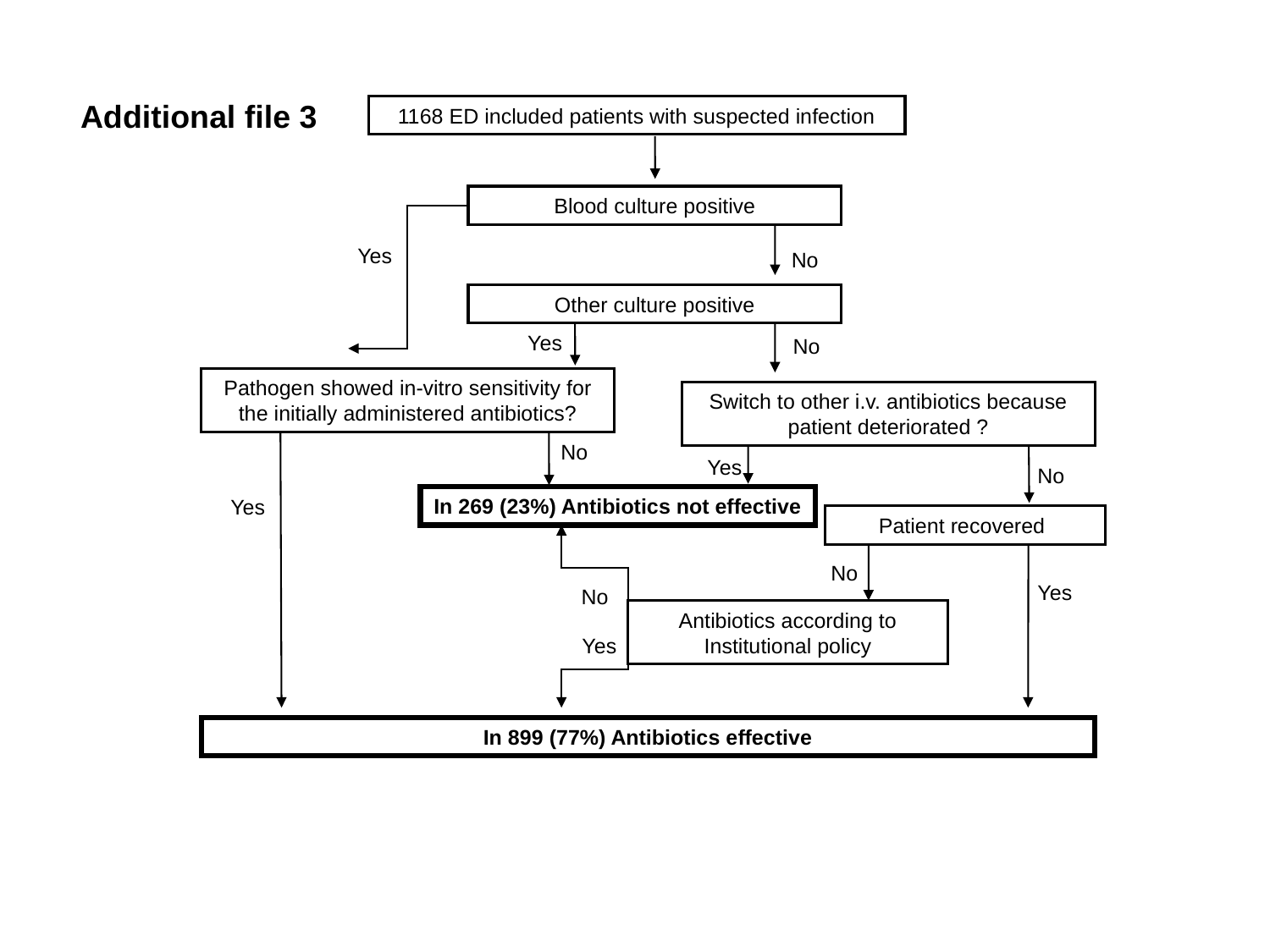

Additional file 3
1168 ED included patients with suspected infection
Blood culture positive
Yes
No
Other culture positive
Yes
No
Pathogen showed in-vitro sensitivity for the initially administered antibiotics?
Switch to other i.v. antibiotics because patient deteriorated ?
No
Yes
No
In 269 (23%) Antibiotics not effective
Yes
Patient recovered
No
Yes
No
Antibiotics according to Institutional policy
Yes
In 899 (77%) Antibiotics effective
